# Non-communicable Diseases Week: Best Practices in Addressing the NCDs Burden from Tanzania

**DOI:** 10.5334/aogh.4116

**Published:** 2023-12-15

**Authors:** Belinda J. Njiro, Jackline E. Ngowi, Harrieth P. Ndumwa, Davis Amani, Castory Munishi, Doreen Mloka, Emmanuel Balandya, Paschal Rugajo, Anna T. Kessy, Omary Ubuguyu, Bakari Salum, Appolinary Kamuhabwa, Kaushik Ramaiya, Bruno F. Sunguya, Erick A. Mboya, Amani I. Kikula, Emilia Kitambala, James Kiologwe, James T. Kengia, Ntuli Kapologwe

**Affiliations:** 1 School of Public Health and Social Sciences, Muhimbili University of Health and Allied Sciences, Dar es Salaam, Tanzania; 2 School of Pharmacy, Muhimbili University of Health and Allied Sciences, Dar es Salaam, Tanzania; 3 School of Medicine, Muhimbili University of Health and Allied Sciences, Dar es Salaam, Tanzania; 4 Ministry of Health, Tanzania; 5 President’s Office Regional Administration and Local Government, Tanzania; 6 Tanzania Non-Communicable Diseases Alliance, Tanzania; 7 Tanzania Diabetes Association, Tanzania; 8 Shree Hindu Mandal Hospital, Tanzania; 9 Muhimbili University of Health and Allied Sciences (MUHAS), Tanzania

**Keywords:** non-communicable diseases (NCDs), NCDs week, NCDs advocacy, Tanzania

## Abstract

**Background:**

Five million people die every year from non-communicable diseases (NCDs) globally. In Tanzania, more than two-thirds of deaths are NCD-related. The country is investing in preventive and advocacy activities as well as interventions to reduce the burden. Of particular interest, the Ministry of Health (MoH) commemorates NCDs’ week using a multisectoral and multi-stakeholders’ approach. This paper highlights activities conducted during NCDs week with the aim of sharing lessons for other countries with similar context and burdens.

**Methods:**

A thorough review of official reports and the national strategic plans for NCDs was done including the 2020 and 2021 National NCDs’ week reports, the National Strategic Plan for NCDs 2015–2020, and the National NCDs agenda.

**Findings:**

NCDs week is commemorated annually throughout the country involving the five key activities. First, community awareness and participation are encouraged through media engagement and community-based preventive and advocacy activities. Second, physical activities and sports festivals are implemented with a focus on developing and renovating infrastructures for sports and recreation. Third, health education is provided in schools to promote healthy behaviors for secondary school adolescents in transition to adulthood. Fourth, health service provision and exhibitions are conducted involving screening for hypertension, diabetes, obesity, alcohol use, and physical activities. The targeted screening of NCDs identified 10% of individuals with at least one NCD in 2020. In 2021, a third of all screened individuals were newly diagnosed with hypertension, and 3% were found to have raised blood glucose levels. Fifth, the national NCDs scientific conferences conducted within the NCDs week provide an avenue for stakeholders to discuss scientific evidence related to NCDs and recommend strategies to mitigate NCDs burden.

**Conclusion:**

The initiation of NCDs week has been a cornerstone in advocating for NCDs control and prevention in the country. It has created awareness on NCDs, encourage healthy lifestyles and regular screening for NCDs. The multi-stakeholder and multi-sectoral approaches have made the implementation of the mentioned activities feasible and impactful. This has set an example for the united efforts toward NCD control and prevention at national, regional, and global platforms while considering contextual factors during adoption and implementation.

## Background

Noncommunicable diseases (NCDs) cost more than five million lives annually. This is equivalent to 74% of all deaths globally [1]. At least three in every four NCDs related deaths are reported from the lower- and middle-income countries (LMIC) [2]. These regions are still facing unprecedented toll from infectious diseases, burdening their health systems. This current surge of NCDs highlights the double burden of communicable and non-communicable diseases especially in poorer and marginalized communities [3]. Annually, cardiovascular diseases account for most deaths (17.9 million), followed by cancer (9.3 million), respiratory diseases (4.1 million) and diabetes (1.5 million) [1].

In Tanzania, NCDs account for 34% of all deaths; more than half are due to Hypertension and cancers alone [4]. In 2018 NCDs were responsible for 31% of premature deaths [4]. There is still a gap in the promotion of healthy lifestyles and preventive measures for NCDs, most at-risk population do not know their status [5]. Evidence from rural Tanzania suggests that, a third of adults aged 25–64 years in rural Tanzania were hypertensive and about two-thirds of these were not aware of their hypertension status [5]. Only 35.4% of those known to be hypertensive were on antihypertensive medications [5].

Tanzanian Ministry of Health (MoH), in collaboration with local and international stakeholders, has put in place a number of efforts to curb the growing burden of NCDs. The NCD division with a functional National Steering Committee for NCDs under the MoH was established in 2008 [6]. Also, the launching of the first National NCD Strategy for the prevention of NCDs was done 2009, followed by the establishment of the national NCDI poverty committee in 2016, as among a few initiatives toward addressing NCDs in the country [6,7].

To align with the global action plan for reducing the NCDs burden recommended by the World Health Organization (WHO) [8], the Prime Minister of the United Republic of Tanzania launched the National NCDs Prevention and Control Program in November 2019. The strategy aimed to bring together different sectors to discuss and recommend measures and collaborate in addressing the burden of NCDs in the country. Along with this, the execution of NCDs commemoration week was recommended every second week of November [6]. Organization of NCDs weeks utilized a multi-stakeholder approach with collaboration among various government and non-governmental actors. From the government, there has been a collaboration between the MoH and President’s Office, Regional Administration and Local Governments (PORALG), academic and research institutions and hospitals among other stakeholders. This has created an institutional synergy among the participating institutions in joining efforts to combat the rising burden of NCDs in Tanzania.

There is still a gap in NCDs advocacy, preventive measures and management in LMICs. Despite its proven benefits for early disease identification, management and reducing serious complications, regular and voluntary screening for NCDs is not an in-built habit in Tanzania and most LMICs [9,10]. NCDs week is a useful platform to promote primary and secondary prevention of NCDs through creating awareness on NCDs, community engagement activities, promoting appropriate healthy lifestyles, screening services, and sharing evidence-based practices on NCDs. This paper aims to report on the advocacy and awareness activities conducted during the NCD week for the prevention, control, and management of NCDs in Tanzania; sharing the approach and deriving lessons for other countries.

## Methods

### Design and context

This study used a desk review approach to collect data from published and grey literature, including official national and government reports and documents on NCDs week. Investigators accessed official reports prepared by the organizing committees during the implementation of the second and third NCDs week in 2020 and 2021, respectively. The 2020 and 2021 National NCDs commemoration reports obtained from the Ministry of Health were reviewed on the details of the activities planned and executed during the NCDs week. The authors also reviewed the National Strategic Plan for NCDs 2015–2020 and the National NCDs agenda for references on the strategies, efforts, and plans for NCDs control in Tanzania. A thorough review was also performed to collect data from the published literature on a global and regional picture of NCD advocacy and prevention efforts in other countries. The main sources of information were obtained from WHO and United Nations (UN) websites and reports on global action for NCDs, National NCD alliances, and peer-reviewed articles on preventive and screening services for NCDs. Data synthesis was done narratively and through data visualization methods to present findings from NCD screening activities during NCD commemoration weeks.

### Data recording from the NCD screening activities

Following health promotion and community engagements during NCDs week, all community members were invited to participate voluntarily in the NCDs screening conducted later in the week. All participants who accepted to be screened were recorded manually using standard structured case report forms at the screening booths. A total of 127 healthcare workers, including medical doctors, nurses, medical officers, and laboratory technicians, provided NCD screening services and medical consultations for community members in 18 Tanzanian regions. For each participant screened, a record was kept of their assessment results, diagnoses, and interventions provided. Health Management Information System (HMIS) delegates were also involved in screening activities to monitor health records.

### Data sources and compilation

Each region compiled a report of the health promotion activities implemented and the number of community members reached, screened, and treated for NCDs during the NCDs commemoration week. Data from all participants were anonymized, amalgamated, and kept in the final record in the form of tables and figures. The Ministry of Health compiled reports from all regions to form a national NCDs’ week report.

## Findings

NCDs week has been commemorated annually throughout the country and the event involves five key activities targeting all aspects of NCDs prevention and management. Activities that focused on primary prevention include (i) Community awareness and participation, (ii) Physical activities and sports festivals, and (iii) Health education in schools. Secondary prevention involved (iv) Health service provision and exhibitions and continuity of care for patients with NCDs, and (v) National NCDs scientific conferences covered both primary and secondary prevention aspects. NCD week commemoration activities were implemented in 23/26 (88.5%) Tanzanian regions, reaching a total of 198,000 community members who participated directly in at least one health promotion activity.

### Community awareness and participation

During NCDs week, various media platforms have been used to ensure that the community is reached with necessary information on the control and prevention of NCDs. These include newspapers, television (TV), health institution websites, and community gatherings. This has been implemented through medical experts’ interviews and participatory programs on different media platforms, newspaper editorials, and verticals, and sharing educational content through social media channels.

Community involvement and advocacy were conducted focusing on healthy dietary habits and encouraging age-appropriate physical activities. The community has also been educated about the risk factors that predispose one to NCDs. Moreover, through community-based interventions and screening, individuals are emphasized on the importance of regular preliminary screening to ensure early disease detection and timely initiation of treatment to reduce morbidity and consequences resulting from NCDs.

### Physical activity and sports festival

To emphasize the significance of physical activity in the prevention of NCDs, sports have been widely promoted during NCDs weeks in Tanzania. Several sports activities including netball, volleyball, basketball, and football took place in the form of recreation and competition during the NCDs week sports festivals. Along with different kinds of sports, jogging and cycling were also conducted. The festivals were initiated by joint physical exercises and a walk that included all community members and regional leaders before the sports competitions. The sports activities were officiated by national and regional leaders who also took part and winners for each sports category were awarded medals and trophies by the respective guest of honors during the closing ceremonies. Physical activity being an important component in combating the NCDs, during the NCDs weeks several infrastructures for sports and recreation were developed and renovated. For instance, in 2021, the Mgambo pitch for netball and volleyball was renovated to offer a supportive environment for sports in commemoration of the third NCDs week.

### Health education in schools

In promoting healthy behaviors and practice at all ages, the NCDs week events have not left behind children and adolescents. These efforts have targeted secondary schools’ students who are in the transition period from childhood to adolescence. The MoH in collaboration with other stakeholders were able to make official visits to secondary schools, particularly in the hosting regions; Dodoma, Dar es Salaam and Arusha where they raised awareness among students on NCDs burden, risk factors, and preventive measures to combat it. During the visits, students were actively engaged in sports and interactive educational sessions. Moreover, students’ and teachers’ representatives from the respective schools were allowed to attend the National NCDs scientific conferences. This strengthened the linkage and demonstrated the role of the Ministry of Education in preventing NCDs and reducing the burden thereof by empowering adolescents to develop and adopt to a healthier lifestyle at an early stage. Students were allocated in different conference sessions to learn of the country NCD status, also as the platform for establishing mentorship and linkage with different experts. During the 3^rd^ NCDs week in 2021, students constituted about a third (31.9%) of all individuals (63,161) reached by the NCDs awareness activities.

### Health service provision and exhibitions

Community-based services provided during the NCDs week have been covering mental health, nutrition, cancer, eye and vision care, oral and dental health, accident and prosthetic and emergency care services. Nevertheless, laboratory services and health insurance services were not left on the list. The services have been provided by trained and qualified doctors, nurses, laboratory specialists, medical officers, coordinators of the Health Management Information Systems (HMIS) and health attendants. These services were provided under two arms: community health education and exhibitions and, provision of medical services such as screening, referral and initiation of treatment. Clients were initially tested for blood pressure, body mass index (BMI) and blood glucose level followed by counseling on healthy lifestyle which was more emphasized among those found with risk factors during screening. Clients who were diagnosed to have any of the non-communicable diseases were directed to designated specialists at the site whereas appropriate referrals were provided for further medical evaluation, management and continuity of care. Further counseling on different health issues was provided to all clients in the exhibition areas.

**Figure 1:**
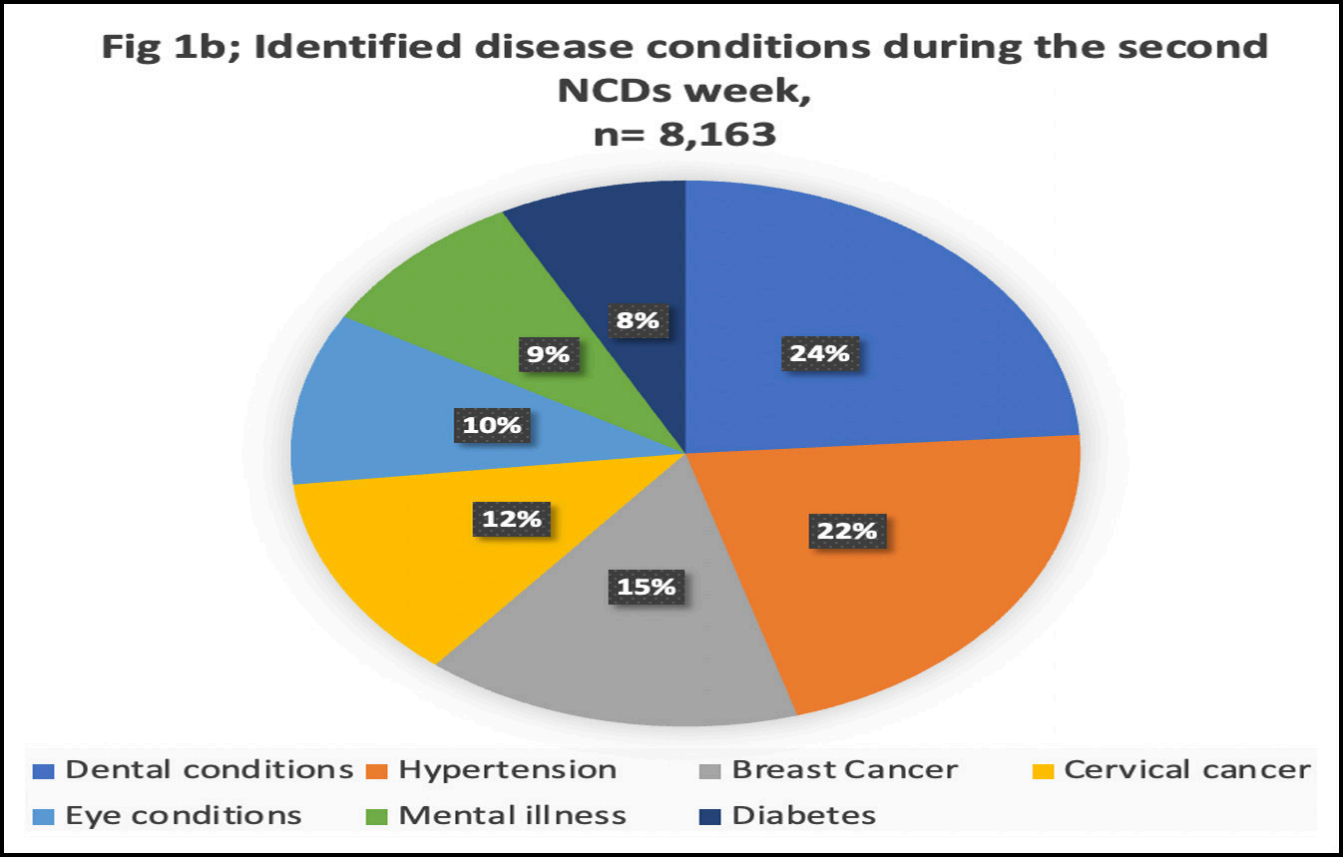
Screening and identification of patients with various non-communicable diseases during the second NCDs commemoration week in 2020.

Through screening services, the NCDs weeks have also been vital to providing data on the status of NCDs in the country as well as the existing modifiable and non-modifiable risk factors. Screening for various health conditions was conducted among 77,959 community members during the second NCDs commemoration week in 2020. The data identified 10% (8,163/77,959) of screened individuals to have one or more medical conditions. Dental conditions (24%), hypertension (15%), breast (15%) and cervical cancers (12%) were the most prevalent NCDs identified during screening; the distribution of the specific disease conditions is shown in Figure 1.

**Figure 2:**
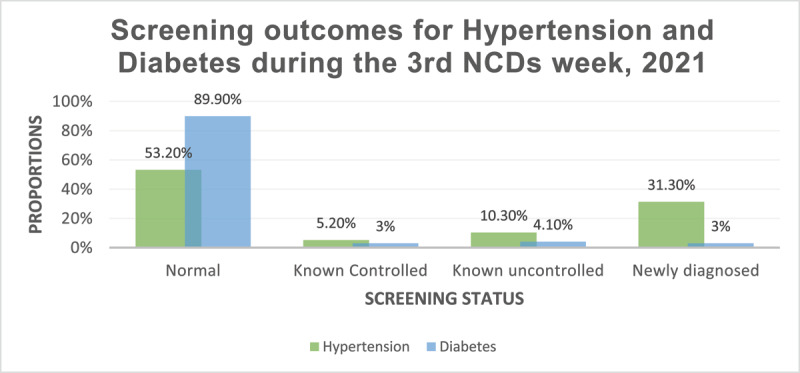
Screening outcomes for patients screened for hypertension and diabetes mellitus during the third NCDs week in 2021.

In 2021, among a total of 2030 individuals screened for hypertension, 950 (46%) had high blood pressure; of these, about a third (31%) were newly diagnosed, and 15% were known hypertensive, either controlled on medications or uncontrolled. During the same period, we screened a total of 2026 individuals for diabetes; 10.1% of these had raised blood glucose, with newly diagnosed individuals comprising 3% of these (Figure 2).

**Figure 3:**
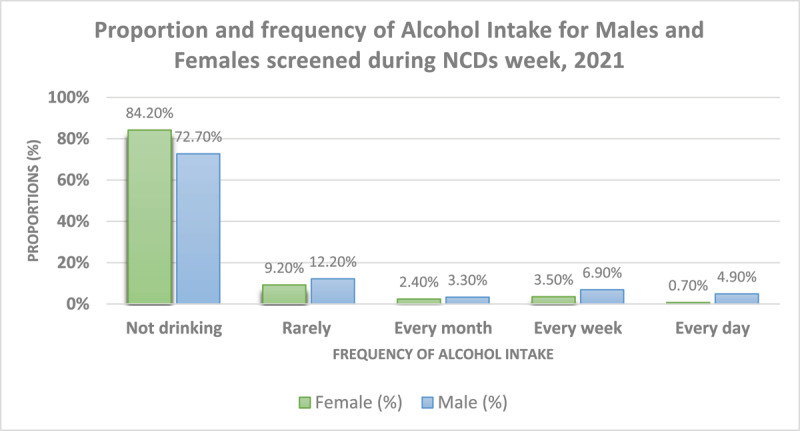
Proportions of self-reported alcohol intake stratified by sex during the third NCDs week in 2021.

A total of 1472 people were also screened for obesity and about one third (29.6%) of the people did not meet the WHO-recommended 150 minutes of physical activity per week. Moreover, 35% of individuals reported not taking fruits and vegetables for five or more days per week. About 16% of females and 27% of males screened reported alcohol intake; with 5% of males reporting daily alcohol intake (Figure 3).

### Scientific Conferences

The NCDs week events in the country have been concluded by the NCDs conferences which provide a platform for stakeholders to discuss “scientific” issues related to NCDs. In the efforts to curb the growing burden of NCDs globally, a number of conferences, meetings and congresses on NCDs were organized and the outcomes and recommendations from these scientific works have been crucial in bringing up strategies and policies for NCDs control and prevention. A total of 3 national NCDs scientific conferences have been successfully conducted in three different regions. The first conference was held in 2019 at the University of Dodoma, the second in 2020 at Julius Nyerere International Convention Center in Dar es Salaam, and the third one in 2021 was held at Arusha International Conference Center. Through these conferences, recommendations and evidence-based practices were presented to the government and other relevant stakeholders contributing to the efforts and strategies for addressing the high burden of NCDs in the country.

## Discussion

This study summarizes the strategies and activities conducted during the NCDs week also the short-term outcomes following its implementation since 2019. It highlights the role of community involvement activities and their role in preventive strategies against NCDs; also, in the early detection of disease and linkage to care. Integrating NCD screening during the annual NCDs week was shown to be feasible and can increase the enrolment of previously unidentified patients into available health services. As reported elsewhere, promoting early detection and early enrolment into treatment is vital in preventing fatal complications [10,11]. Furthermore, preventive measures are known to be more cost-effective and reduce the cost of health financing for NCDs to individuals and the health system [12].

The annual NCDs commemoration week in Tanzania prioritized community engagement activities; the educational and screening strategies were community-centered to ensure that people take charge of their health. This has been supported by Abimbola et al., whose research shows results in favor of community engagement for health, with community-led strategy yielding more favorable outcomes for NCDs prevention [13]. Involving intended beneficiaries and other relevant stakeholders throughout the process of promoting interventions is also recommended [14].

In an effort to reduce the burden of non-communicable diseases in the country, the focus has shifted towards prevention by raising awareness through various media platforms. According to Tabassum et al., more awareness and reliable sources of information on NCDs are needed to ensure the directed efforts toward prevention and early management [15]. A consolidated approach and sustained effort to raise awareness of the preventive measures through media engagement were proposed as the most interactive and feasible method of spreading information on NCDs and promoting healthy lifestyles [8,16,17].

Health promotion and prevention activities are the most cost-effective and feasible methods to reduce the NCDs burden in LMICs [10,12]. During the NCDs commemoration weeks, the community was also reached and encouraged to participate in screening for the common NCDs. The targeted screening identified 10% of individuals with at least one NCD in 2020. In 2021, a third of all screened individuals were newly diagnosed with hypertension and 3% were found to have raised blood glucose levels. These activities are in support of the WHO NCDs prevention and control policy; given the significance of early management in reducing NCDs-related mortality, a call is made to ensure the availability and accessibility of screening interventions for NCDs in LMICs where the burden is relatively higher [10,18].

About two-thirds of individuals who attended the screening activities reported not engaging in sufficient levels of physical activities as recommended. Studies have shown that sport is a gateway to physical activity and healthy lifestyles [19]. Sports as physical activity helps in reducing the risk factor of non-communicable diseases and promotes general well-being [20]. Through the sports festivals and jogging clubs, the NCDs week heightened the significance of physical activities as a preventive measure by leading by example.

To ensure the provision of age-appropriate awareness for NCDs, school educational programs on NCDs were among the strategies implemented during the NCDs weeks in Tanzania. When children and adolescents are exposed to NCDs risk factors, they often suffer severe health consequences with an increased likelihood of developing complications. Therefore, primary prevention programs must target this population to promote healthy adulthood [21]. Moreover, the WHO recommends that the education sector holds a fundamental responsibility to protect children’s health and well-being by promoting healthy eating and physical activity at all levels of education, establishing standards for meals provided in schools, and incorporating quality physical activity into the daily curriculum for schools among others [22].

Organization of NCDs week in Tanzania has brought sensitization in all regions in Tanzania to organize screening services for NCDs such as diabetes, hypertension, cancer, mental health, nutrition, and dental health, among others. This is a new trend that did not exist before and has been useful in identifying people with such conditions, providing them with counseling, treatment, and appropriate referrals for further management and care.

Implementing NCDs week in Tanzania has faced some challenges. First, incomplete documentation and record keeping, particularly during screening activities, have made it difficult to note the NCDs’ burden trends following advocacy activities. Only 23 of 26 regions compiled a report of the activities implemented; out of these, only 18/23 reported the number of community members reached in screening activities. Most data reported during the advocacy and screening activities were recorded manually, hence prone to incompleteness and human error. Secondly, the efforts made to implement preventive and advocacy activities differ among regions. Some regions were able to reach a bigger population in terms of NCDs advocacy and screening as compared to others. Thirdly, delays in compiling and sharing reports by some regions hindered early implementation of the recommendations and NCDs policy changes. The fourth challenge was brought by threats of COVID-19 and restrictions on holding gatherings, specifically during the third NCD week in 2020 when preparations for the NCDs week were delayed. Additionally, other governmental activities in 2020 such as the general election of the President and members of parliament challenged the planning process. Several stakeholders could not participate and thus brought a great challenge to the availability of resources required for preparations and the provision of services. The most affected aspect was the NCD conference, where the team required at least six months to prepare and organize the conference. Lastly, Financial and human resources for health have been a striking challenge in implementing the objectives of the NCDs weeks. The inadequate number of health care workers to conduct NCDs screening and limited availability of diagnostics equipment and supplies were also barriers to providing services to clients during the NCDs week.

Our report should be interpreted in the light of a few limitations. We used secondary data in this research, which may limit the provision of full details on the commemoration activities. We have summarized data from the Ministry of Health reports that were initially prepared for purposes other than research, which may have introduced bias in reporting our findings.

## Conclusions and Recommendation

The initiation of NCDs week has been a cornerstone in advocating for NCDs control and prevention in the country. The main focus has been to create awareness on NCDs, encourage healthy lifestyles and regular screening for NCDs among Tanzanians as a significant proportion of people present late during the course of the disease, leading to serious complications. The events have brought together key stakeholders on NCDs, provision of NCDs screening services and improvement of infrastructures and facilities such as the renovation of hospitals and sports grounds.

Moreover, NCDs week events have provided an opportunity for scientists, policymakers and other stakeholders to convene, share and discuss various scientific findings with policy and practice implications. To ensure sustainability and improvement of such impactful activities in addressing NCDs in the country, we recommend integrating NCDs with other pre-existing programs for communicable diseases such as malaria, HIV and TB. This will also ensure effective utilization of both financial and human resources given the challenge of limited resources in LMICs.

Ensuring continuity of care following screening and referral for newly diagnosed clients is crucial and should be among the priorities. The impact of screening activities will not be realized if there is no linkage to care; the referrals made and follow-up for patients must be owned by the targeted communities. Data collection and documentation is another useful aspect worth attention for continued evaluation and keeping track of the progress made for future reference.

Overall, the national NCDs weeks in Tanzania have marked one of the best practices for controlling and preventing NCDs in the country and if similar efforts are modified and implemented in other settings, it might lead to united efforts toward NCDs control and prevention in national, regional and global platforms. Nevertheless, aligning with the WHO global action for NCDs prevention and control, we recommend taking considerations of the contextual factors during the adoption and implementation of the NCDs week in other countries.
